# Screening and Application of Ligninolytic Microbial Consortia to Enhance Aerobic Degradation of Solid Digestate

**DOI:** 10.3390/microorganisms10020277

**Published:** 2022-01-25

**Authors:** Ulysse Brémond, Aude Bertrandias, Jérôme Hamelin, Kim Milferstedt, Valérie Bru-Adan, Jean-Philippe Steyer, Nicolas Bernet, Hélène Carrere

**Affiliations:** 1 Air Liquide, Innovation Campus Paris, 1 Chemin de la Porte des Loges, 78354 Jouy-en-Josas, France; ulysse.bremond@airliquide.com (U.B.); aude.bertrandias@airliquide.com (A.B.); 2 INRAE, Université de Montpellier, LBE, 102 Avenue des Étangs, 11100 Narbonne, France; jerome.hamelin@inrae.fr (J.H.); kim.milferstedt@inrae.fr (K.M.); valerie.bru@inrae.fr (V.B.-A.); Jean-Philippe.Steyer@inrae.fr (J.-P.S.); nicolas.bernet@inrae.fr (N.B.)

**Keywords:** anaerobic digestion, biogas, solid digestate, aerobic consortia, lignin, bioaugmentation

## Abstract

Recirculation of solid digestate through digesters has been demonstrated to be a potential simple strategy to increase continuous stirred-tank reactor biogas plant efficiency. This study extended this earlier work and investigated solid digestate post-treatment using liquid isolated ligninolytic aerobic consortia in order to increase methane recovery during the recirculation. Based on sampling in several natural environments, an enrichment and selection method was implemented using a Lab-scale Automated and Multiplexed (an)Aerobic Chemostat system to generate ligninolytic aerobic consortia. Then, obtained consortia were further cultivated under liquid form in bottles. Chitinophagia bacteria and Sordariomycetes fungi were the two dominant classes of microorganisms enriched through these steps. Finally, these consortia where mixed with the solid digestate before a short-term aerobic post-treatment. However, consortia addition did not increase the efficiency of aerobic post-treatment of solid digestate and lower methane yields were obtained in comparison to the untreated control. The main reason identified is the respiration of easily degradable fractions (e.g., sugars, proteins, amorphous cellulose) by the selected consortia. Thus, this paper highlights the difficulties of constraining microbial consortia to sole ligninolytic activities on complex feedstock, such as solid digestate, that does not only contain lignocellulosic structures.

## 1. Introduction

Anaerobic digestion is an established biological process that allows the conversion of organic matter into biogas, a renewable energy, and digestate, a natural fertilizer. The biogas sector may grow importantly in the coming years as the availability of biomass to produce these gases is enormous and largely unused [1]. However, the sector remains strongly dependent on governmental incentives as the production cost of biogas is higher than for fossil gas [2]. Therefore, it is important to find strategies to reduce production cost and improve the economic feasibility of the field.

It has been shown that various pathways exist to recover additional energy from the digestate, as some biodegradable organic matter generally remains [3]. One of the simplest strategies is recirculating the solid fraction of digestate, obtained after phase separation, back inside the digester. By doing so, the refractory and complex solid organic matter that composed the solid digestate can remain longer under anaerobic conditions, allowing further conversion to biogas. Without any post-treatment and by recirculation, the methane production of a continuous stirred-tank reactor biogas plant can be increased between 0.6 and 6.3% [4]. In the same study, short-term aerobic post-treatment of the solid digestate (SD) using endogenous microorganisms was not successful and led to lower methane potentials due to carbon losses via respiration. Nonspecific activities towards the lignin-like fraction were identified as the main cause for these results. Nevertheless, many studies showed that aerobic pretreatment of agricultural lignocellulosic feedstocks (straw, stover, residues) can be enhanced by the addition of liquid hydrolytic microbial consortia [5]. These microbial consortia were isolated from natural environments (compost, manure, rotten sawdust) and showed high lignocellulosic activities under aerobic conditions [6,7,8,9].

Experiments were performed to obtain a liquid consortium of ligninolytic microorganisms that can be spread on the SD to degrade specifically the lignin-like fraction of SD during the subsequent short-term aerobic post-treatment. It was hypothesized that efficiency of short-term aerobic post-treatment will be increased and that the residual methane potential of SD will be enhanced. To test this hypothesis, a four-step experiment was performed: (i) sampling organic materials in various environments of interest; (ii) enrichment and selection of ligninolytic microorganisms using an innovative system called Lab-scale Automated and Multiplexed (an)Aerobic Chemostat system (LAMACs); (iii) recovery and cultivation of these microorganisms in suspension; (iv) application of obtained consortia to SD during short-term aerobic post-treatment.

The objective of this study was to answer the following questions: (i) Is selection pressure towards ligninolytic activities really carried out via the designed strategy? (ii) Are the initial inocula from the natural environments becoming more similar after such treatment? We then evaluated the capacity of the selected consortia to increase the efficiency of short-term aerobic post-treatment of SD. In this paper, based on these two parts, the interest in this strategy for SD post-treatment is also discussed.

## 2. Materials and Methods

### 2.1. Initial Environments

Samples from six natural environments were taken to select ligninolytic consortia. Three of them were sampled in the National Nature Reserve of the Massane Forest (Argelès-sur-mer, France). This environment is particularly rich in dead wood as no forestry operation has been allowed since its classification in 1973. Three samples were obtained in the forest: soil with wood decomposition (F1), deep forest litter (F2) and rotten wood (F3). Fresh sheep rumen (RU) was obtained from the INRAE experimental domain of la Fage (Le Viala-du-Pas-de-Jaux, France), following gut sampling on the sheep livestock according to a standardized method using a gastroesophageal tube and a vacuum pump [10]. Rotten wheat straw (RS) was obtained from a field of a farm located in the Gers department (France). Finally, a commercial granulated organic fertilizer (CF) from a composting process was used. The samples were transported to the laboratory at room temperature and immediately used to conserve endogenous microbial communities.

Solid digestate used in this study came from an agricultural biogas plant treating sequential crops, cattle manure, beet pulp, cereal dust and whey. More information on this plant can be found in Brémond et al., under the name Biogas Plant A [4].

### 2.2. LAMACs—Enrichment and Selection

LAMACs is a system that was originally designed to perform continuous anaerobic digestion in a large number of repeated experiments in parallel [11]. A great advantage of this system is its modularity and flexibility. It allows up to 30 chemostats to be operated under aerobic conditions or reactors to be to connected to each other to run them in series. These features were used for the experiment. Pictures of the LAMACs system and a detailed scheme of the experimental set-up are shown in Figure S1.

A LAMACs module is made of six 250 mL glass reactors, eighteen peristaltic pumps (FZ10, A2V, Gazernan, France) with the associated controller module (TMCM 6110, Trinamic, Hamburg, Germany), a custom-made heating block (Garaud, Carcassonne, France), and a magnetic stirring plate (Variomag Multipoint 6, Thermo Scientific, Waltham, MA, USA). For each environmental sample, three 250 mL reactors were used. The first reactor, R1, was used to obtain an enriched consortium. Effluent from this reactor was equally split and continuously introduced into the second reactor (R2) and third reactor 3 (R3). R2 and R3 contained a lignin-rich carbon sources, i.e., 5 g of wheat straw (3 to 8 cm pieces) and 15 g of untreated chestnut woodchips (3 to 5 cm pieces), respectively. In total, three LAMACs modules were used with eighteen reactors run in parallel.

The set-up comprising three reactors, containing a magnetic stirrer in R1, 5 g of wheat straw in R2, and 15 g of chestnut wood in R3, and already connected to each other, were sterilized (121 °C, 30 min). Then, 20 g in the case of solids (F1, F2, F3, CF and RS) or 20 mL for liquid (RU) of initial environment were added aseptically into R1. Then, initial environments were diluted as all R1 reactors were topped up with sterile “enrichment solution” to 200 mL. R1 contains only as a carbon source Kraft lignin at 1 g/L. Kraft lignin was previously successfully used to enrich liquid medium containing soil with lignin-degrading microorganisms [12]. The enrichment solution is based on a salt M9 media recipe made of Na_2_HPO_4_ at 6 g∙L^−1^, KH_2_PO_4_ at 3 g∙L^−1^, NaCl at 0.5 g∙L^−1^, at NH_4_Cl 1 g∙L^−1^ and 1 mL∙L^−1^ of a solution of oligoelements made of FeCl_2_ at 2 g∙L^−1^; CoCl_2_ at 0.5 g∙L^−1^; MnCl_2_ at 0.1 g∙L^−1^; NiCl_2_ at 0.1 g∙L^−1^; ZnCl_2_ at 0.05 g∙L^−1^; H_3_BO_3_ at 0.05 g∙L^−1^; Na_2_SeO_3_ at 0.05 g∙L^−1^; CuCl_2_ at 0.04 g∙L^−1^ and Na_2_MoO_4_ at 0.01 g∙L^−1^. Finally, this solution was complemented with yeast nitrogen base at 0.1 g∙L^−1^ and 0.01 mL∙L^−1^ of vitamin solutions (RPMI-1640, Sigma-Aldrich, Darmstadt, Germany). Oligoelements and vitamins were added to avoid growth limitations. The solution was sterilized via filtration through 0.2 µm cellulose acetate filter or autoclaving (30 min, 121 °C).

The reactors were placed in the heating block of the LAMACs. Sterile bags of 250 mL (Easyflex+, Macopharma, Mouvaux, France) filled in with sterile “enrichment media” and a 5 L bottle containing sterile M9 media (salts and oligoelements only, following the recipe given above without any carbon source) were connected to each set-up. Heating blocs were set at 30 °C. The stirring speed was set at 150 rpm for R1 reactors. Aquarium pumps were also connected and used to inject continuously filtered air (0.45 µm cellulose acetate filter) into each reactor to avoid any oxygen shortage. Finally, custom-made software was used to control all peristaltic pumps (calibrated beforehand) interfacing with free software (TMCL-IDE, Trinamic, Hamburg, Germany). This was the starting point of the enrichment and selection step.

To enrich and then select, the operation of R1 reactors differed from the R2 and R3 reactors:

(i) In R1 reactors, aerobic enrichment was carried out under a liquid state. Initial environments were diluted with the enrichment solution (ratio 1/10 *w*/*w*) to favor the development of lignin-degrading microorganisms that would be able to thrive in liquid [12]. An HRT of 10 days was set to avoid any preselection in ligninolytic microorganisms based on their growth rate. Every day, 20 mL of fresh enrichment solution was pumped into R1, and 20 mL of “enriched solution” was pumped out. Of this volume, 10 mL was pumped out the system and discarded, whereas from the remaining volume, 5 mL was pumped into each of the reactors R2 and R3.
(ii) In R2 and R3 reactors, aerobic lignin-degrading bacteria were selected under a solid state. Wheat straw and chestnut wood were only partially emerged as the liquid content was set to 50 mL. Enriched liquids, from R1 reactors, before being injected in R2 and R3, were ten times diluted with M9 media (45 mL supplied by another peristaltic pump). The HRT in this vessel was 1 day, as 50 mL of liquid was pumped out and discarded every day. The aim of all these conditions was to select microorganisms, coming from liquid media, that are able to fix themselves quickly and irreversibly (to avoid washout due to the short HRT) to wood and straw, and use them as the main carbon source (dilution aims to reduce Kraft lignin concentration). Finally, these reactors were shaken manually once a week to ensure that all wood and straw were in contact with the pumped-in liquid.

The duration of this experiment step was 130 days for F1, F2 and F3 forest environments and 96 days for the other RS, RU and CF environments, because all initial environments were not sampled at the same moment. It was assumed that, after three months of continuous operation (corresponding to 9 HRT in R1 and 90 HRT in R2 and R3), consortia obtained on wood and straw were stable and this experiment step was stopped.

### 2.3. Consortia Propagation

Following microbial consortia selection on wheat straw and chestnut wood, the aim of this subsequent step was to recover and propagate these consortia in a liquid phase. This liquid phase could be then further used as inoculum for SD before short-term aeration post-treatment. This step can be divided in three parts, which were mainly the preparation of the carbon source and solutions that were used in the Erlenmeyer bottles, the transfer procedure from LAMACs to the Erlenmeyer bottles, and the cultivation and sub-culturing steps.

#### 2.3.1. Preparation of the Carbon Source and Solutions

As a carbon source, a complex sterilized solid digestate was used to already accustom consortia to use the lignin-like fraction. SD was complexified via a chemical method described in Jimenez et al., (2015) [13]. Composition of the SD used in this study was characterized previously via the same methodology [4]. Raw SD had the following composition in percentage of the total COD: 5% ± 0.1 soluble sugars and proteins, 10.8% ± 0.1 remaining proteins and sugars, some humic substances and lipids, 39.8% ± 0.1 hemicellulose and cellulose and 44.3% ± 0.3 lignin-like compounds.

Frozen SD was first dried at 45 °C. Then, SD was mixed with 0.1 M sodium hydroxide solution for 1 h at room temperature (100 g of dry SD in 5 L solution). Then, supernatant was removed via centrifugation. SD pellets were recovered and put back in a fresh 0.1 M sodium hydroxide solution. This step was repeated 5 times in order to completely remove the most easily degradable fractions from the SD. At the end of this step, only cellulose, hemicellulose and lignin-like compounds remained. Finally, the remaining SD pellets obtained were dried at 45 °C and aliquoted in hermetically closed plastic containers (3 g per container). All plastic containers filled with SD were then sterilized by gamma irradiation by Ionisos (Dagneux, France). Complex SD was subject to a cycle of successive gamma ray sterilization with an average applied radiation dose of 73.6 kGy. Such a dose was estimated to be sufficient regarding the quantity of bacteria in SD (internal measurements give values up to 10^12^ cells g^−1^ for raw SD before sterilization). Gamma ray sterilization was preferred to autoclaving to keep the organic matter structure undisturbed. Then, sterilized complex SD was used as a carbon source for propagation of selected consortia by Erlenmeyer cultivation.

The solution used in the Erlenmeyers was based on a mineral medium recipe [14]. This solution contains all necessary elements for microorganisms to grow (except carbon). Demineralized water was completed with: Na_2_HPO_4_·2H_2_0 at 3.5 g∙L^−1^; KH_2_PO_4_ at 1 g∙L^−1^; (NH_4_)_2_SO_4_ at 0.5 g∙L^−1^; MgCl_2_ 6H_2_0 at 0.1 g∙L^−1^; CaCl_2_ at 0.1 g∙L^−1^, and 1 mL∙L^−1^ of a solution of oligoelements made of FeSO_4_·7H_2_0 at 0.2 g∙L^−1^; CoCl_2_ 6H_2_0 at 20 mg∙L^−1^; ZnSO_4_ 7H_2_0 at 10 mg∙L^−1^; MnCl_2_ at 5 mg∙L^−1^; Na_2_MoO_4_.2H_2_0 at 3 mg∙L^−1^; Na_2_SeO_3_ at 2 mg∙L^−1^; NiCl_2_·6H_2_0 at 2 mg∙L^−1^ and CuCl_2_ 2H_2_0 at 1 mg∙L^−1^. This solution was autoclaved (30 min, 121 °C). The measured pH of the solution was 7.2.

#### 2.3.2. Transfer Procedure

LAMACS set-ups were disassembled and liquid phases in R2 and R3 reactors were totally purged via pumping. Subsequently, remaining wheat straw (R2 reactor) and chestnut wood (R3 reactor) were washed using 60 mL of a sterile phosphate buffer solution (PBS) and Zirconium Oxide ceramic beads of 6.35 mm diameter (MP Biomedicals, Santa Ana, CA, USA) were used to detach and recover into solution as many microorganisms as possible that had grown on the surface. PBS was made of demineralized water completed with NaCl at 2.28 g∙L^−1^, Na_2_HPO_4_ at 0.306 g∙L^−1^ as well as NaH_2_PO_4_ at 0.108 g∙L^−1^ and autoclaved for 30 min at 121 °C. A quantity of 60 mL of this solution and ceramic beads were added in each R2 and R3 reactor, which were subsequently agitated one time manually for two minutes. In total, twelve solutions (six from straw and six from wood) were obtained. These solutions were further used as microbial inocula for Erlenmeyer propagation.

#### 2.3.3. Propagation in Liquid Phase

Baffled Erlenmeyer flasks (Duran Schott, Mainz, Germany) of 250 mL, supplied with a screw cap composed of a 0.2 µm PTFE autoclavable membrane, were used for aerobic consortia propagation. These flasks were autoclaved, then filled under sterile conditions with 100 mL of sterile mineral medium, then 25 mL of enriched PBS solution (source of microorganisms) was poured in, and finally 3 g of sterile complex SD (source of carbon) was added into the solution. Then, caps were closed with a 0.2 µm PTFE membrane allowing air circulation, and flasks were placed for a duration of 40 days on a Stuart SSL1 orbital shaker (Cole-Parmer, Vernon Hills, IL, USA). The room temperature was regulated at 24 °C and continuous agitation was set at 120 rpm, to ensure proper mixing of solutions and SD. Only one Erlenmeyer per environment was prepared.

In this step, four additional types of Erlenmeyer bottle were prepared and placed under the same conditions (agitation, temperature), to answer several hypotheses/research questions:

(i) Two Erlenmeyer flasks where only sterile complex SD was used and no source of microorganisms was added. These were the negative controls and aimed to check the sterility of the complex SD used as the carbon source in this propagation experiment. These are referred to as “Sterility-check” samples.
(ii) Erlenmeyer flasks where the source of microorganisms was fresh SD. Practically, fresh SD was washed with sterile PBS (2 g of TS in 50 mL of PBS). Then this liquid was poured in the same proportion (25 mL) as previously in three flasks containing sterile complex SD and minimal mineral media. These were positive controls that allowed measurement of the endogenous activity of microorganisms of SD during this propagation experiment. These are referred to as “Dig” samples.
(iii) Erlenmeyer flasks where the source of microorganisms was fresh dry compost fertilizer (CF). Practically, fresh CF was washed with sterile PBS (2 g of TS in 50 mL of PBS). Then, this liquid was poured in the same proportion (25 mL) as previously in three flasks containing sterile complex SD and minimal mineral media. These were controls that allowed measurement of the activity of microorganisms from CF without any enrichment and selection steps using LAMACs. These are referred to as “Direct-CF” samples.
(iv) Erlenmeyer flasks where the source of microorganisms was a quick selection of endogenous SD microorganisms able to degrade Kraft lignin. Practically, fresh SD was washed with sterile PBS (2 g of TS in 50 mL of PBS). Then, this liquid was poured under sterile conditions into a dozen Petri dishes containing M9 medium complemented with Kraft lignin at 1 g∙L^−1^ and Agar at 15 g∙L^−1^. After one week at 30 °C, the biggest colonies were harvested and mixed directly into the minimal mineral medium. It was then supplemented in three flasks with sterile complex SD. These were positive controls that allowed the measurement of the activity of endogenous microorganisms of SD, quickly selected on Petri dishes (instead of the LAMACs system), after their culture in Erlenmeyer flasks. These are referred to as “Petri” samples.

An overview of this experiment step, in addition to the five different types of Erlenmeyer flask that were prepared, is given in the Supplemental File (Figure S2).

### 2.4. Addition of a Consortia Solution to Enhance Short-Term Aerobic Post-Treatment

After 40 days in the Erlenmeyer flasks, consortium propagation was stopped. To screen the obtained consortia on their capacity to enhance efficiency of short-term aeration post-treatment of SD, the following protocol was applied. This was the last experiment step.

First, to only evaluate the action of the liquid consortia and not endogenous SD microorganisms, we used as a substrate a sterile SD. Fresh SD was dried at 45 °C and then sterilized by gamma irradiation according to the same procedure as for complex SD (see Section 2.3.1). Here, easy-to-degrade fractions of SD were not chemically removed to fully evaluate the capacity of liquid consortia to specifically degrade the most complex fractions. Then, 1.5 g TS of sterile SD was placed in 575 mL biomethane potential flasks and 11 mL of liquid consortia from Erlenmeyer flasks was added. Such a ratio allows a good contact between the liquid consortia and the SD matter, the latter being slightly emerged in the liquid. For each liquid consortium, flasks in triplicate were prepared. In addition, a blank was added, where liquid consortia were replaced by water. Finally, flasks remained open and were placed in an incubator at 30 °C (similar temperature as for the LAMACs step) without agitation for a 6-day duration corresponding to a short-term aerobic post-treatment.

At the end of this period, biomethane potential (BMP) tests were launched directly using the flasks according to the protocol described in Bremond et al., (2021) [4]. A control made of untreated sterilized SD was added to the BMP run. In total, 17 different types of short-term aerobic post-treatment were tested, depending on the type of consortia used (LAMACs ×12, Sterility-check, Dig, Direct-CF, Petri and water). Methane production obtained from BMP tests was expressed as a function of the initial amount of SD placed in the BMP flasks. Therefore, carbon losses due to respiration were taken into account despite the fact that they were not measured during the post-treatment. Finally, chemical oxygen demand (COD) measurements were performed on liquid consortia to evaluate additional methane that could be produced during the BMP test due to remaining organic matter in the liquid [4]. Figure S3 in the Supplemental File describes in detail this last step. In addition, Table 1 provides a comprehensive overview of the different experimental conditions and steps of this study.

### 2.5. Microbial Community Analysis

The following sampling 1procedures were applied for liquid and solids:

(i) Liquid samples were centrifuged. Supernatants were eliminated and remaining pellets were further stored at −20 °C before use.
(ii) For solid sampling, they were stored in 2 mL Eppendorf tubes (generally between 0.1 and 0.5 g of solid samples) at −20 °C before genomic DNA extraction.

Microbial sampling was carried out at three different moments of the experiment:

(i) The six initial environments were sampled before their use in R1 reactors of LAMACs. For F1, F2, F3, CF and RS, this corresponded to a solid sampling, whereas for RU it was a liquid sampling.
(ii) At the end of the LAMACs step, liquid sampling was performed on the twelve solutions obtained after PBS solid washing of wheat straw and chestnut wood, for each of the six initial environments. Liquid solutions were also sampled following the PBS washing step of solids used for the additional conditions tested (Dig, Direct-CF, Petri). In addition, the Sterility-check sample consisted of a solid sample of sterilized SD.
(iii) Finally, at the end of the Erlenmeyer step, liquid sampling was carried out on all Erlenmeyer flasks that were used as inoculate for the short-term aerobic post-treatment.

Subsequently, DNA was extracted, purified and PCR-amplified for sequencing. Moreover, qPCR was performed on all these samples for bacteria and eukaryotes. A detailed protocol for each of these steps is available in the Supplemental File (see Supplementary Methods S1). Sequencing results were further processed using the R packages hillDiv [15] and Phyloseq [16]. The first was used to analyze alpha and beta diversities over the course of the experiment. The second package was used to display evolution of the dominant OTUs over the different experiment steps. A detailed description of the choices made for these analyses is available in the Supplemental File (see Supplementary Methods S2). Finally, qPCR results were used to evaluate microbial growth over the different experiment steps. DNA quantity per vessel was determined by combining qPCR results and the associated quantity of solids or liquid present in the vessel. The DNA quantity in all vessels used in the different experiment steps was thus measured.

## 3. Results

### 3.1. Alpha Diversity Analysis

**Figure 1:**
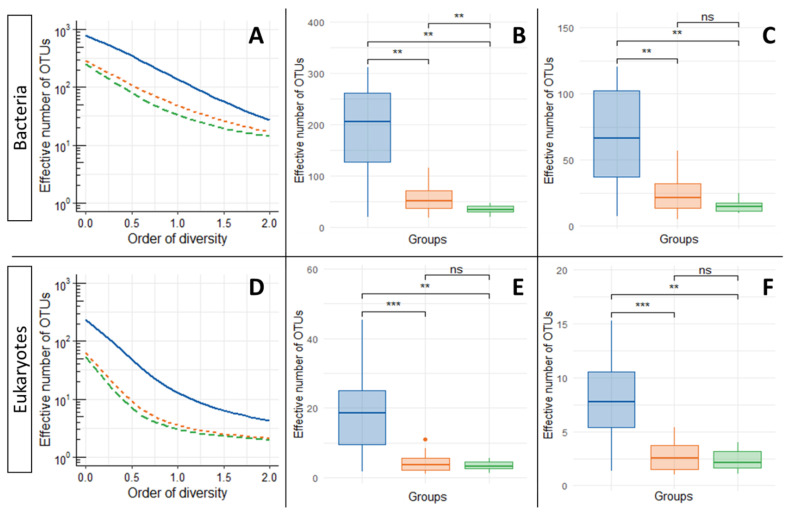
(**A**) Alpha diversity profile of bacteria population for an order of diversity (q) varying between 0 and 2; (**B**) boxplot comparison of bacteria Hill numbers for q = 1 (Shannon equivalent); (**C**) boxplot comparison of bacteria Hill numbers for q = 2 (Simpson equivalent); (**D**) alpha diversity profile of eukaryotes’ population for a q varying between 0 and 2; (**E**) boxplot comparison of eukaryotes’ Hill numbers for q = 1 (Shannon equivalent); (**F**) boxplot comparison of eukaryotes’ Hill numbers for q = 2 (Simpson equivalent). For all sub-figures: solid blue lines and blue boxplot correspond to initial samples; dotted orange lines and orange boxplot correspond to samples recovered at the end of the LAMACs step; dashed green lines and green boxplot correspond to samples recovered at the end of the propagation step. Box plot pairwise median comparison was performed using a Wilcoxon rank-sum test, the following scale was used to indicate significance test result: ns: *p* value > 0.05; **: *p* value ≤ 0.01; ***: *p* value ≤ 0.001.

Evolution of alpha diversity for bacteria and eukaryotes over the experiment steps is presented in Figure 1. For bacteria and eukaryotes, the average alpha diversity profile significantly decreases between initial environments and samples recovered at the end of the LAMACs or propagation step. For bacteria, the initial median Shannon diversity (Hill parameter q = 1) value of 205 effective number of OTUs significantly drops to 50 after the LAMACs step and then to 34 (also significant) after the propagation step. For Simpson diversity (Hill parameter q = 2), corresponding to dominant OTUs, the initial median value of 66 effective number of OTUs significantly drops to 21 after the LAMACs step and to 15 after the propagation step. A similar trend was observed for eukaryotes. The initial median Shannon diversity value of 19 significantly drops to 4 after the LAMACs step and to 3 after the propagation step. For Simpson diversity, the initial median diversity value of 8 significantly drops to 2 after the LAMACs or propagation steps. Only compost fertilizer (CF) did not show a reduction in its diversity (lowest value of all initial environment box plots). This can be explained by the fact that this sample comes from an industrial composting process that has already applied a selective pressure on microbial flora, leading to a low initial diversity in comparison to samples originating from natural environments (i.e., forests, etc.).

Regarding these results, it can be concluded that LAMACs steps efficiently applied a selective pressure on five of the six tested environments, reducing initial microbial diversity. A greater contrast was found for the impact of the propagation step on diversity. For the eukaryotes, no significant change was observed, indicating that diversity is maintained over time. This is in accordance with our initial strategy, as the aim of this step was mainly to propagate selected consortia and not particularly to apply an additional selective pressure. However, for bacteria, a significant drop was observed for q = 1, which can be due to the shift from a solid-state environment in LAMACs to a liquid cultivation in Erlenmeyers, in addition to the use of another source of carbon (complex SD). Additional selective pressure is not necessarily a negative fact because, in this case, it was directed towards our aim: to obtain a liquid consortium able to use complex fraction of SD.

### 3.2. Beta Diversity Analysis

**Figure 2:**
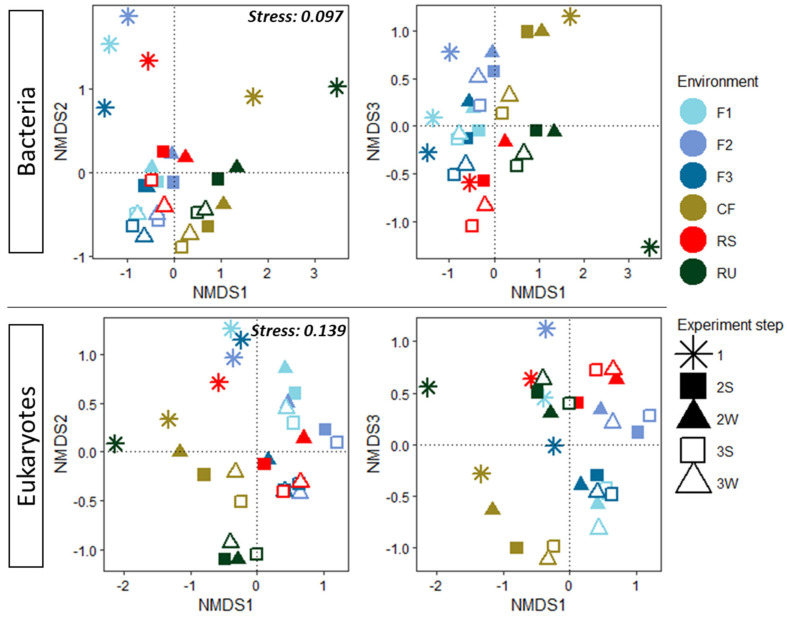
Bacteria and eukaryotes’ NMDS representation of Horn dissimilarity matrix for all environments and experiment steps. Environments: soil with wood decomposition (F1), deep forest litter (F2), rotten wood (F3), commercial granulated organic fertilizer (CF), rotten wheat straw (RS), fresh sheep rumen (RU). Experiment steps: (1) Initial environment; (2S) LAMACs straw; (2W) LAMACs wood; (3S) Propagation straw; (3W) Propagation wood.

Similarly to the previous section, samples that were enriched and selected via the LAMACs process were analyzed. It was decided to base this analysis on Horn distance (q = 1) because it is a trade-off between rare and dominant species, and thus provides a fair visualization of beta diversity. An NMDS method, presented in Figure 2, was implemented to represent the main distance relationships among all the samples.

For bacteria and eukaryotes, NMDS were plotted in three dimensions to reach a reliable representation. For bacteria, a low ordination stress value (0.097) indicates that representation in three dimensions is excellent. It can be seen that, after the LAMACs step, all initial environments become closer and seem to converge to a central point in that representation. This means that initial bacterial communities are becoming more similar after LAMACs. This higher similarity is maintained over the propagation step as samples remain closer to each other. For eukaryotes, a relatively low ordination stress value (0.139) indicates that representation in three dimensions is good. However, in that representation, initial eukaryote communities do not seem to become more similar after LAMACs or propagation steps, because samples do not display any clear convergence pattern.

Thus, microbial communities from initial environments become more similar after LAMACs and propagation steps, whereas this does not appear to be the case for eukaryotes. One potential explanation may lie in the fact that, for the six initial environments, the effective number of OTUs for bacteria is, on average, always almost one log above that for eukaryotes (see Figure 1). Therefore, the initial reduced number of eukaryotes species may lower the probability of having shared species between the different environments that would be positively selected by the LAMACs step.

Based on the further analysis of beta diversity described in the Supplemental File (see Figure S4 and associated explanations), an additional conclusion is that the system type (LAMACs, Erlenmeyers) drives the microbial community evolution more than the carbon sources used for selection (straw and wood).

### 3.3. Microbial Communities: Composition and Quantity over Experiment Steps

**Figure 3:**
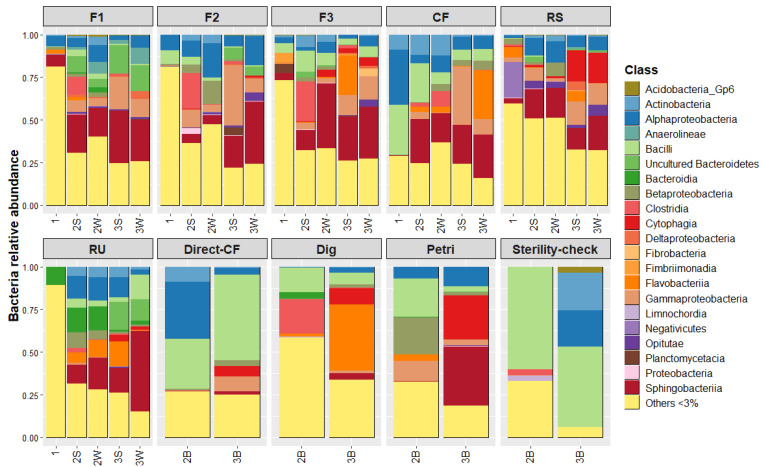
Evolution of bacterial communities over experiment steps as a function of the initial environment. Experiment steps’ distinction within each initial environment follows this nomenclature: (1) Initial environment; (2B) Blank initial environment; (2S) LAMACs straw; (2W) LAMACs wood; (3B) Propagation blank (3S) Propagation straw; (3W) Propagation wood.

**Figure 4:**
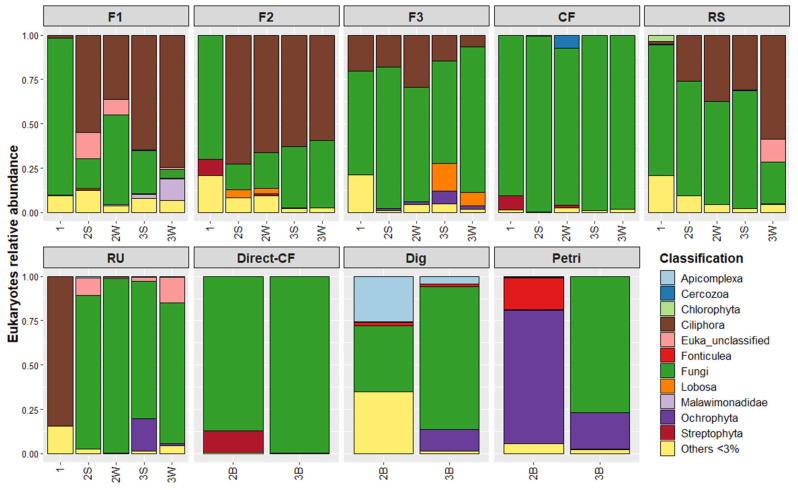
Evolution of eukaryotic communities over experiment steps as a function of the initial environment. Experiment steps’ distinction within each initial environment follows this nomenclature: (1) Initial environment; (2B) Blank initial environment; (2S) LAMACs straw; (2W) LAMACs wood; (3B) Propagation blank (3S) Propagation straw; (3W) Propagation wood.

Relative abundance of microbial communities as a function of initial environments and environment steps are displayed in Figure 3 for bacteria and in Figure 4 for eukaryotes. Blank microbial communities are also displayed (Direct-CF, Dig, Petri and Sterility-check). It can be noted that it was not possible to amplify eukaryotes’ DNA for sequencing in the case of the two Sterility-check samples. Therefore, sequencing data are not presented for these samples.

Several observations can be made for bacteria when looking at Figure 3: (i) The CF sample is indeed less diverse than the five others. The initial amount of minor OTUs (<3% relative abundance) is around 30%, whereas others are comprised of between 63 and 88%. (ii) For F1, F2, F3, RS and RU initial environments, up to 65% (for RU) reduction in the percentage of minor OTUs through experiment steps can be observed. (iii) The effect of LAMACs is underlined by the Direct-CF blank, as its bacterial community after propagation was mainly dominated by bacilli that is different from the CF environment after LAMACs, which is dominated by Flavobacteria, Gammaproteobacteria and Sphingobacteriia. (iv) Petri dish selection on Kraft lignin efficiently decreased the quantity of minor OTUs. In addition, Sphingobacteriia was the dominant class after propagation of the Petri sample. (v) Finally, the Sterility-check blank showed a shift in its microbial composition after the propagation step, which means that there were still some remaining living bacteria despite the strong sterilization procedure. Actinobacteria and Alphaproteobacteria were the two major classes that developed during the propagation step. However, the profile of the Sterility-check blank is very different from that of all other propagation samples. Dominant Bacilli, Actinobacteria and Alphaproteobacteria for Sterility-check do not exceed 25% of total relative abundance in propagation samples that were supplemented with a source of microorganisms (except for Direct-CF, which was almost 60%). Therefore, it can be assumed that when an additional source of microorganisms was added to the propagation system, development of remaining endogenous SD bacteria had a limited impact on the final bacteria composition.

Similarly, Figure 4 allows several observations to be drawn concerning eukaryotes: (i) The percentage of minor OTUs is extremely low (7%, on average, for all samples), even for initial environments in comparison to bacteria. (ii) For the six initial environments, fungi dominate in forest, compost fertilizer and rotten straw samples, whereas sheep rumen is dominated by Ciliophora. This difference can be explained by the fact that rumen is a liquid medium rich in bacteria favorable to the development of Ciliophora, which are mainly bacteria predators. (iii) There is no clear trend in eukaryotes’ evolution over the experiment steps for the six environments. For F1, F2 and RS, the initial dominant fungi dropped in favor of Ciliophora; for F3 and CF, the initial dominant fungi remain; and for RU, the dominant Ciliophora are replaced by fungi. This may explain why no convergence was observed for eukaryotes in Figure 2. (iv) Direct-CF displays, after the propagation step, a profile very similar to that of CF (almost 100% fungi), showing that the LAMACs step has no clear effect on eukaryotes for an environment with an already low diversity profile. (v) Finally, selection on a Petri dish of endogenous SD microorganisms favors Ochrophyta and Fonticulea eukaryotes over fungi and Apicomplexa (for Dig). However, in the Petri sample after the propagation step, Fonticulea and Ochrophyta were strongly reduced in favor of fungi. The final composition was very similar to that of the Dig sample. Therefore, it appears that the effect of the Petri dish is erased by the propagation step.

**Figure 5:**
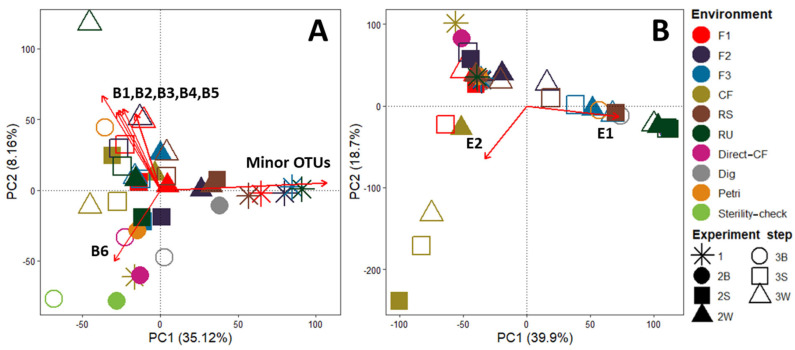
Principal component analysis based on relative abundance of bacteria OTUs (**A**) and eukaryotes’ OTUs (**B**). The red arrows were obtained via Envfit analysis and indicate significant OTUs (*p* value < 0.01). B1 to B6, and E1 and E2, correspond to bacteria or eukaryotes that are further described in Table 2. Experiment steps’ distinction within each initial environment follows this nomenclature: (1) Initial environment; (2B) Blank initial environment; (2S) LAMACs straw; (2W) LAMACs wood; (3B) Propagation blank (3S) Propagation straw; (3W) Propagation wood.

One conclusion that emerges from these observations is that there is no clear trend regarding the type of microorganisms that were selected over these experimental steps. Therefore, an additional data visualization was required to obtain insight into these microorganisms. This corresponds to Figure 5, which was based on PCA representation of the OTUs table and the application of the Envfit analysis.

For bacteria, the first dimension represents around 35% of the total variability and allows samples rich in minor OTUs to be distinguished. Most initial environments are rich in minor OTUs (except CF as previously stated), and LAMACs and propagation steps successively reduce their amount in favor of dominant species. The second dimension axis explains 8% of the total variability and six OTUs (B1 to B6) were determined as significant variables. Table 2 provides a detailed taxonomy of these bacteria. First, Dig, Petri and Sterility-check samples after the propagation step can be distinguished from the other samples and were enriched in Paenibacillus. These are facultative anaerobic or strictly aerobic bacteria known to be able to hydrolyze a variety of carbohydrates (Carboxymethyl cellulose, xylan, starch, chitin, etc.) by releasing extracellular carbohydrases [17]. For selected samples via LAMACs or Petri dish, five OTUs appear to have been enriched after the propagation step. Four of these (B1 to B4) are Bacteroidetes and one is a Firmicute of the genus Paenibacillus (B5). Bacteroidetes are reported to be, in general, efficient degraders of complex carbohydrates [18]. Three of the four are from the Chitinophagia class (ex-Sphingobacteriia) and two of them are part of the Chitinophagacea family. In this family, bacteria are reported to be aerobic or facultatively anaerobic and are often found in soils. In addition, some species are reported to be able to degrade chitin polymers or cellulose [19,20].

For eukaryotes, the first two dimensions represent almost 60% of the total variability. Here, for 9 of the 12 samples from the six initial tested environments and subjected to LAMACs and propagation steps, contents of Sordariomycetes were significantly increased. This was also the case for Dig and Petri samples after the propagation step. Sordariomycetes are Ascomycota fungi commonly found in soils and decaying wood, and in aquatic environments [21]. Most of these are reported to be able to break down lignin and cellulose from plant debris. In this study, liquid state cultivation during the propagation step may have potentially favored Sordariomycetes growth.

**Figure 6:**
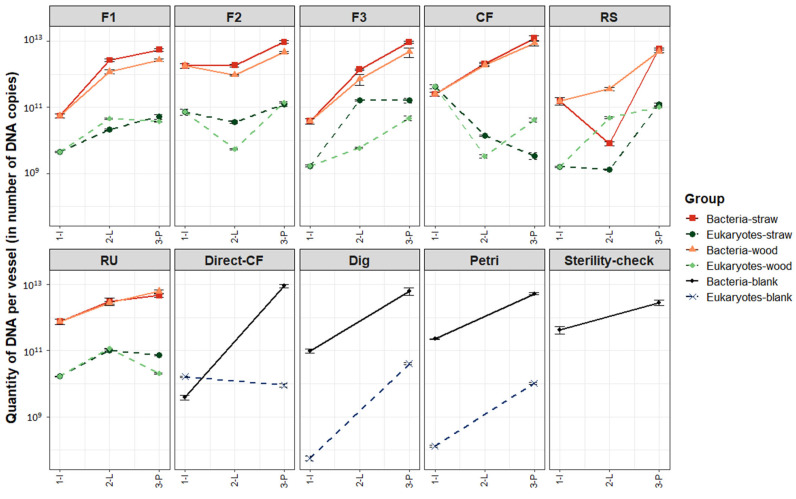
Evolution of vessel DNA quantities as a function of the experiment step and the initial environment. Bacteria and eukaryotes were separated into different lines. Experiment steps’ distinction within each initial environment follows nomenclature: (1-I) Initial environment; (2-L) End LAMACs step; (3-P) End propagation step.

Finally, we checked if LAMACs and propagation steps led to microbial growth over time. The DNA quantity was calculated based on qPCR results in each reactor or flask. These quantities are presented in Figure 6 as a function of the initial environment and the experiment step.

For bacteria, DNA quantities after the propagation step ranged from 2.9 × 10^12^ 16S copies (Sterility-check) to 1.2 × 10^13^ 16S copies (CF). DNA increased for all initial environments and blanks. This increase happened to a larger extent for F1, F3, CF, RS, Direct-CF, Dig and Petri (more than a log of difference) than for F2, RU and Sterility-check (less than a log of difference). However, for these latter three, initial bacteria DNA quantities were already high (close to 10^12^) in comparison to the other environments. For F1, F2 and F3, slightly more DNA were obtained at the end for samples that went through LAMACs selection on straw than samples selected on wood. For CF, RS and RU, the final amount was identical in samples of straw and wood.

For eukaryotes, DNA quantity after the propagation step ranged from 1.4 × 10^10^ 18S copies (CF-straw) to 1.6 × 10^11^ 18S copies (F3-straw). Evolution of DNA quantity seems to converge towards this quantity range. Indeed, DNA quantity was increased for environments with initial DNA quantities below 5 × 10^9^ (F1, F3, RS, RS, Dig and Petri). DNA quantities were stable for environments having initial DNA quantities already within the final region (F2, RU and Direct-CF). A decrease in DNA quantity happened for the CF initial sample that had a high DNA quantity in comparison to all other samples (4.2 × 10^11^).

Thus, except for CF eukaryotes, quantities of DNA in Erlenmeyer bottles at the end of the propagation step were increased both for bacteria and eukaryotes’ species in comparison to initial enrichment reactors (R1). This result is positive because it shows that the microbial communities were active and grew throughout the experiment steps.

### 3.4. Evaluation of the Impact of Consortia Addition on Efficiency of Short-Term Aerobic Post-Treatment—Ecosystem Function Relationship

**Figure 7:**
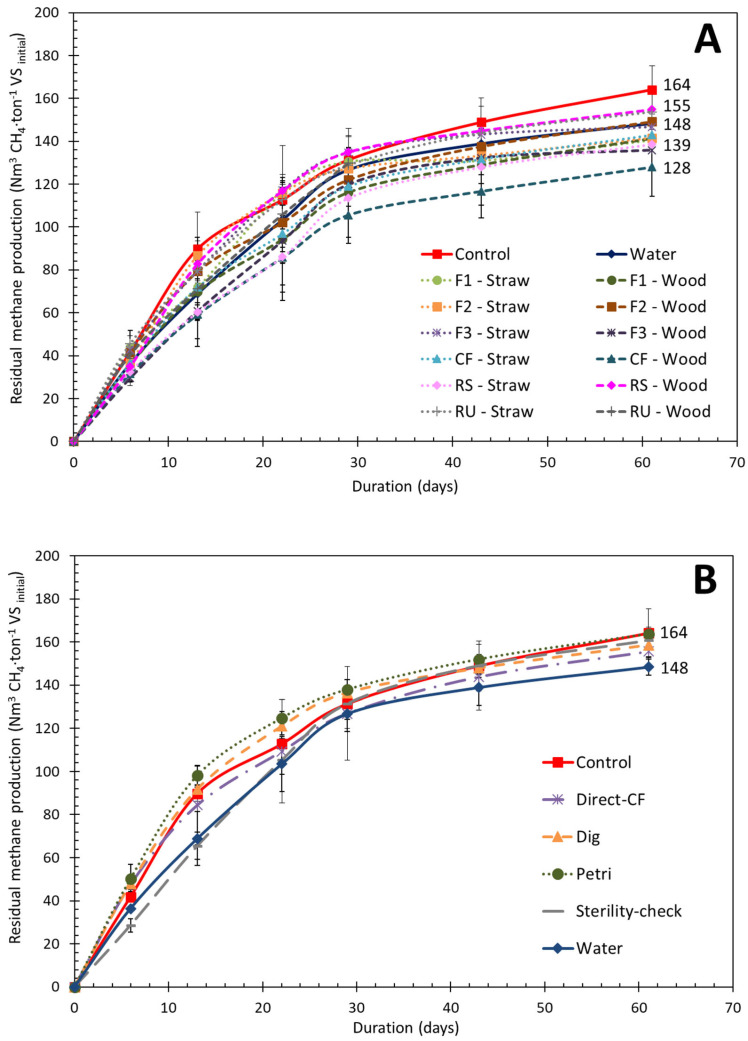
Residual methane potential curves from BMP tests; (**A**) mostly shows BMP test results from SD treated with consortium solutions coming from the LAMACs, whereas (**B**) only shows BMP test results from SD treated with blanks.

Figure 7 displays BMP test results for sterilized SD that were subject to short-term aeration post-treatment with selected consortia or blank solutions obtained from the propagation step. First, the BMP value of the control (164 ± 11 Nm^3^ CH_4_.ton^−1^ VS) is in the range of values found in the literature (60–240 Nm^3^ CH_4_.ton^−1^ VS), showing that drying and sterilization had no significant effect on methane potential [3]. Water blank (water added to the dry and sterile SD instead of consortia before the 6-day incubation) is significantly 10% lower than control (148 Nm^3^ CH_4_.ton^−1^ VS_initial_). This shows that remaining endogenous microorganisms consume organic carbon and still do not display specific ligninolytic activities, as previously observed [4].

After short-term aerobic treatment with addition of consortia selected on straw, the average BMP was 144 ± 7 Nm^3^ CH_4_.ton^−1^ VS_initial_. This was significantly lower than the control value, by 14%. For consortia selected on wood, the average BMP value was 143 ± 12 Nm^3^ CH_4_.ton^−1^ VS_initial_, which was not significantly different from the control value. Similarly, all four tested blanks (Direct-CF, Dig, Petri, Sterility-check) had BMP between 156 and 164 Nm^3^ CH_4_.ton^−1^ VS_initial_, and were not significantly different from the control.

Here, it is important to underline that the 11 mL of liquid consortia added had a soluble COD that was estimated to give an average maximal theoretical amount of 50 additional mL CH_4_. Because it is a liquid solution, we can assume that anaerobic biodegradability is close to 80% [22]. Therefore, for the inoculated BMP tests, an additional 28 Nm^3^ CH_4_.ton^−1^ VS_initial_dig_ is expected, and the final value should be close to 190 Nm^3^ CH_4_.ton^−1^ VS_initial_dig_. However, none of the BMP tests containing liquid consortia reached this value and the obtained values were even lower or similar to the control.

Based on these considerations, it can be concluded that the selected consortia did not improve the short-term aerobic post-treatment. This is likely to be due to unspecific activities of selected consortia toward the lignin-like fraction of SD. During the aerobic post-treatment, respiration of easy-to-degrade fractions (e.g., sugars, proteins, amorphous cellulose) occurred, leading to a lower methane yield in comparison to untreated SD.

## 4. Discussion

In this study, we implemented a means to select aerobic consortia that potentially display specific ligninolytic activities. However, ecosystem function relationship trials clearly indicate that the obtained liquid consortia were not able to specifically degrade the lignin-like fraction of SD during a short-term aerobic treatment. Several reasons were identified that may explain such results:

(i) Microbial lignin degradation requires a multiplicity of oxidative enzymes and heterogeneous small molecule co-factors that are produced by ligninolytic fungi and bacteria [23]. Currently, lignin biodegradation in nature is thought to occur in two main stages that consist of its depolymerization followed by the mineralization of resultant heterogeneous aromatics [24]. Although bacteria are reported to be dominant and the most active in the mineralization step, this is not the case for the depolymerization step [25]. Indeed, filamentous Basidiomycetes white-rot fungi were identified as major actors in this step due to their capacity to produce high quantities of various oxidative enzymes (e.g., laccase and lignin peroxidase) [26]. In comparison to fungi, the identified ligninolytic bacteria (e.g., *Pseudomonas* sp., *Rhodococcus* sp.) have significantly lower activities during the depolymerization step [24]. For this study, depolymerization of the SD lignin-like fraction is sought because aromatic units can be converted to methane during anaerobic digestion [27]. Assuming that most depolymerization activity is due to dominant microbial species, it is interesting to look at those that were enriched through the experiment steps. For Sordariomycetes fungi and, in general, for Ascomycete, it is reported that the lignin depolymerization rate is slower than for white-rot fungi due to the difference in enzymatic systems (e.g., lack of ligninolytic Class II enzymes) [28]. More generally, fungi obtained after the propagation steps were mostly Ascomycetes. Basidiomycetes that were notably present in the initial forest environments disappeared after the LAMACs step. One hypothesis is that the liquid state during enrichment with Kraft lignin (R1) favored the growth of Ascomycetes over Basidiomycetes. Indeed, Basidiomycetes, due to their filamentous nature, are reported to grow better during solid state fermentation [29]. For bacteria, *Paenibacillus* sp. used in a consortium treating pulp and paper wastewater were reported to be able to degrade and metabolize the higher molecular weight lignin molecules [30]. However, with the exceptions of RU, Direct-CF and Sterility-check, their abundances were relatively low. Finally, Chitinophagia, the most significantly enriched bacteria, are not ligninolytic strains. Instead, their enzymatic activities are directed toward carbohydrate degradation (e.g., deconstruction of dead fungal material via chitin hydrolysis) [20,31]. Thus, dominant fungi and bacteria that were enriched through this experiment are not reported as efficient actors in lignin depolymerization, notably in comparison to white-rot fungi.
(ii) The designed approach to obtain consortia was unconventional in comparison to other existing studies focusing on screening of microbial ligninolytic consortia. Successive transfer with Erlenmeyer flasks or sequential batch reactors containing the targeted final lignin rich substrate are often preferred [32,33,34]. With these kinds of cultivation strategies, it is possible, notably during transfer steps, to perform precise monitoring of the ligninolytic activity (microbial sampling, enzymatic activity, percentage of degraded lignin, etc.). However, with LAMACs, a system that operates in a continuous mode, reactors were closed and such activity monitoring of solids was not possible. It was thus not possible to determine during the experiment if long-term LAMACs operations were sufficient to obtain reduced, stable and ligninolytic-active microbial communities. Afterwards, it appeared that it was not the case and earlier monitoring would have been beneficial to further select consortia.
(iii) From our knowledge, studies on microbial ligninolytic consortia use either classical lignocellulosic biomasses, such as straw or wood, or industrial lignin derivatives [29,32]. In both cases, cellulose, hemicellulose and lignin are the main molecules present. In comparison to these lignin-rich substrates, SD composition is more diverse and contains higher quantities of proteins, sugars, and lipids, which may provide an opportunity for microbial activities other than ligninolytic ones [35]. Thus, the realization of specific ligninolytic activities is even more challenging because consortia may reorient their activity towards easier-to-degrade fractions. It can be hypothesized that, during the short-term aerobic post-treatment, OTUs present in applied consortia and able to efficiently metabolize proteins, sugars or lipids, may have outcompeted ligninolytic species with slower metabolisms (such as Sordariomycetes or *Paenibacillus*). It would have been interesting to test the obtained consortia on more classical substrates (straw, wood) to only evaluate their ligninolytic activities.

## 5. Conclusions

In this study, a screening method for aerobic ligninolytic consortia was implemented. Within an automated continuous LAMACs system, from six initial environments, microorganisms able to use Kraft lignin were enriched and then were selected based on their capacity to attach quickly and grow on wood or straw. A selective pressure was applied as alpha diversity was reduced. In addition, initial microbial communities appeared to converge towards a common structure as dissimilarity distances decreased. A subsequent propagation step ensured growth under liquid conditions of all selected consortia. After these consecutive steps, Sordariomycetes fungi and Chitinophagia bacteria were the two dominant classes of microorganisms that were significantly enriched in most samples.

Finally, addition of these consortia did not increase the efficiency of short-term aerobic post-treatment of SD. Slow or lack of lignin depolymerization activity for dominant selected microorganisms, difficulty of monitoring the evolution of consortia ligninolytic activities during LAMACs steps, and specificity of the SD substrate (e.g., containing proteins) that may generate competition with other types of enzymatic activities, were identified as potential reasons explaining the lower methane yields obtained in comparison to the untreated control. In future studies, a more precise quantification of the ligninolytic activities (Kraft lignin degradation tests for instance) during the different steps (LAMACs, propagation) will be required to identify the most efficient consortia, in addition to consortia metabolic activity characterization in the presence of digestate (do strong proteolytic activities appear?). In a last step, biomethane potential tests should be performed with digestates pretreated with these specific consortia. Further studies will also need to integrate trials with unsterilized digestate because this will be more representative of the final full-scale application. Interactions between the applied consortia and the endogenous microbial digestate community should be studied. In addition, future studies could also apply pure culture of white-rot fungi. These basidiomycetes should display higher lignin depolymerization capacities and lower side activities (proteolytic) than obtained in the microbial consortia in this study. Therefore, the chances to specifically degrade the lignin-like fraction of SD may be greater.

To conclude, this paper highlights the difficulties of constraining microbial consortia toward specific ligninolytic activities in the presence of a complex substrate, where lignocellulose structure is mixed with easier-to-degrade organic material.

## Figures and Tables

**Table 1:** Overview of the experimental conditions and steps applied; Straw and Wood indicate the type of carbon used for selection in the LAMACs step; the colored boxes indicate that the step was applied to the given source of microorganisms; ×1, ×2 or ×3 indicate the numbers of replicates for each step.

Source of Microorganisms	Acronyms	LAMACs Enrichment & Selection	Propagation Erlenmeyer Flasks	Aerobic Post-Treatment	BMP Tests
**Natural environments**					
Soil with wood decomposition	F1	Straw	×1	×3	×3
		Wood	×1	×3	×3
Deep forest litter	F2	Straw	×1	×3	×3
		Wood	×1	×3	×3
Rotten wood	F3	Straw	×1	×3	×3
		Wood	×1	×3	×3
Fresh sheep rumen	RU	Straw	×1	×3	×3
		Wood	×1	×3	×3
Rotten wheat straw	RS	Straw	×1	×3	×3
		Wood	×1	×3	×3
commercial granulated organic fertilizer	CF	Straw	×1	×3	×3
		Wood	×1	×3	×3
Directly from SD via a washing step	Dig		×2	×3	×3
Isolated from SD via Kraft lignin petri dishes	Petri		×3	×3	×3
Directly from CF fertilizer via a washing step	Direct-CF		×3	×3	×3
Sterilized complex SD	Sterility-check		×3	×3	×3
**Blanks**					
Water added to SD instead of liquid consortia	Water			×3	×3
SD without post-treatment	Control				×3

**Table 2:** Detailed taxonomy of significant OTUs obtained from Envfit analysis; % ID corresponds to the % of identity of the closest relatives in NCBI using BLAST.

	Envfit Pr (>r)	Super Kingdom/Kingdom	Phylum	Class	Genus	Species	% ID
B1	0.001	Bacteria	Bacteroidetes	Chitinophagia (ex-Sphingobacteriia)	*Pseudoflavitalea*	*Pseudoflavitalea* sp.	97.85
B2	0.001	Bacteria	Bacteroidetes	Chitinophagia (ex-Sphingobacteriia)	/	*Uncultured Chitinophagaceae bacterium*	99.46
B3	0.007	Bacteria	Bacteroidetes	Chitinophagia (ex-Sphingobacteriia)	*Terrimonas*	*Uncultured Terrimonas* sp.	98.39
B4	0.003	Bacteria	Bacteroidetes	/	/	*Uncultured Bacteroidetes bacterium*	96.12
B5	0.008	Bacteria	Firmicutes	Bacilli	*Paenibacillus*	*Paenibacillus* sp.	97.33
B6	0.009	Bacteria	Firmicutes	Bacilli	*Paenibacillus*	*Enrichment culture clone LDC-5*	99.47
E1	0.001	Eukaryota/Fungi	Ascomycota	Sordariomycetes	*Scopulariopsis*	*Scopulariopsis* sp.	98.76
E2	0.003	Eukaryota/Fungi	Ascomycota	Sordariomycetes	*Acremonium*	*Acremonium* sp.	98.55

## Data Availability

Please contact Correspondence author for data sharing.

## Supplementary Materials

The following supporting information can be downloaded at: https://www.mdpi.com/article/10.3390/microorganisms10020277/s1, Figure S1: (A) General view of a LAMACs module with reactor localization for one initial environment; (B) Picture of the three modules used; (C) Picture of two modules, displaying 12 running reactors (4 initial environments); (D) Components and functioning of one set-up made of three reactors, Figure S2: (A) Overview of the preparation of the Erlenmeyer flasks and the five different sources of microorganisms that were used for the propagation step; (B) Reactor R3 coming from CF before the PBS washing step; (C) Reactor R2 coming from F1 before the PBS washing step, Figure S3: Short-term aerobic post-treatment step overview, Figure S4: Beta diversity ordination plots resulting from a Principal Coordinates Analysis (PCoA) based on Horn distance (q = 1) between samples coming from a similar initial environment. Beta diversity of bacteria corresponds to the upper raw and beta diversity of eukaryotes is presented in the lower raw. A distinction is made in all these plots between the different experiment step, Supplementary Methods 1: Extraction and purification, Real-time quantitative polymerase chain reaction (qPCR), PCR amplification and sequencing, Supplementary Methods 2: Alpha diversity measurement, Beta diversity measurement, Dominant OTUs and microbial growth over time.
