# Supplementary material for: Screening and Application of Ligninolytic Microbial Consortia to Enhance Aerobic Degradation of Solid Digestate
Source: Microorganisms. 2022 Jan 25;10(2):277. doi: 10.3390/microorganisms10020277 (PMC8878073; doi:10.3390/microorganisms10020277)
Supplement: Supplementary file 1 [file microorganisms-10-00277-s001.zip › microorganisms-1554570-supplementary.pdf]

Supplemental File

Screening and application of ligninolytic microbial consortia to enhance aerobic degradation of solid digestate

Ulysse Brémond[a,b], Aude Bertrandias[a], Jérôme Hamelin[b], Kim Milferstedt[b], Valérie Bru-Adan[b], Jean-Philippe Steyer[b], Nicolas Bernet[b], Hélène Carrere[b,*]

[a] Air Liquide, Campus Innovation Paris, 1 Chemin de la Porte des Loges, 78354 Jouy-en-Josas, France

[b] INRAE, Univ Montpellier, LBE, 102 Avenue des étangs, 11100 Narbonne, France

*Corresponding author.
E-mail address: helene.carrere@inrae.fr (H. Carrere).

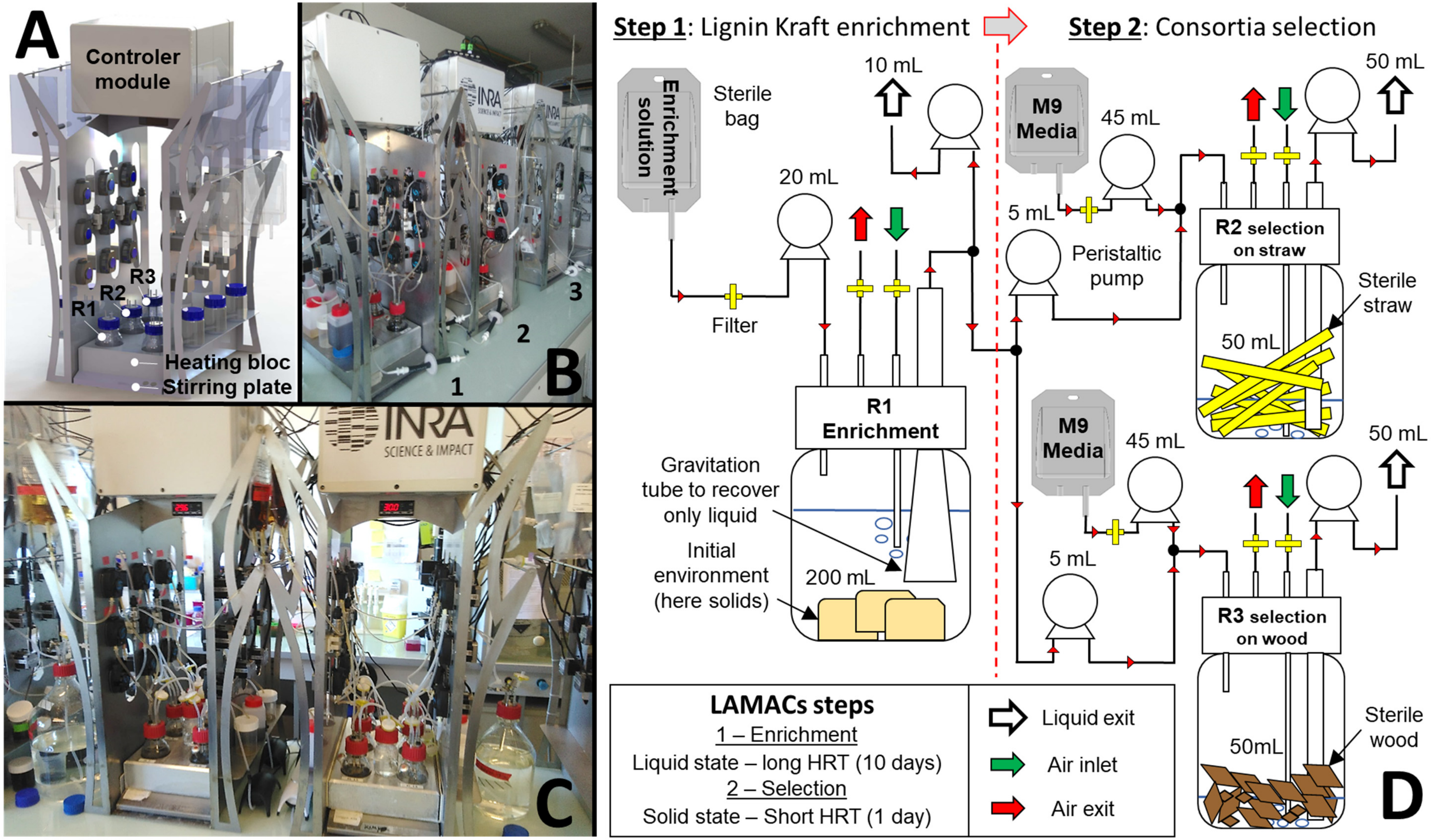

Figure S1. (A) General view of a LAMACs module with reactor localization for one initial environment; (B) Picture of the three modules used; (C) Picture of two modules, displaying 12 running reactors (4 initial environments); (D) Components and functioning of one set-up made of three reactors.

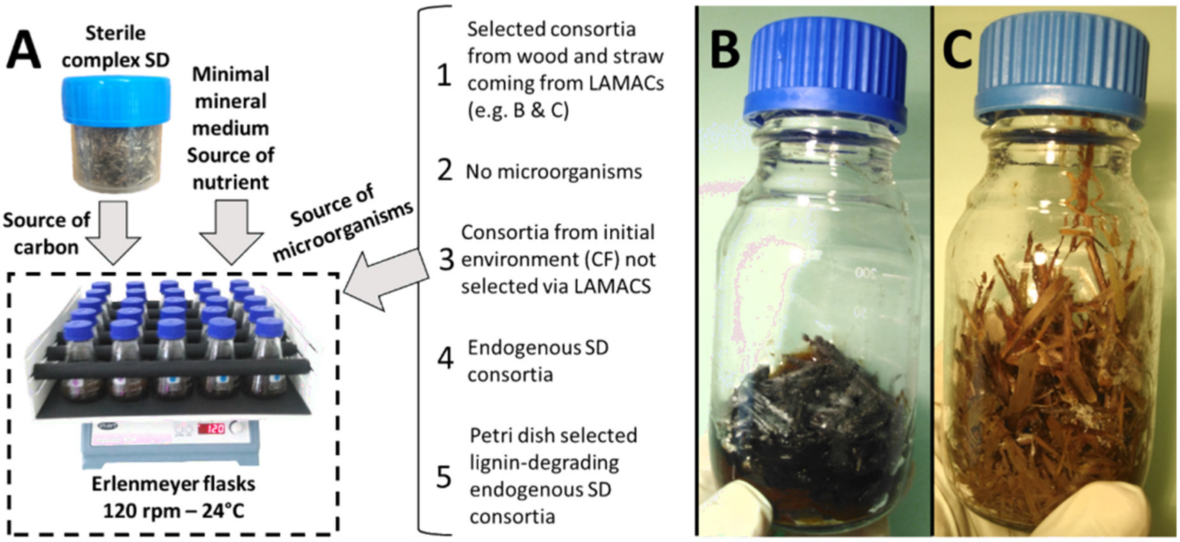

Figure S2. **(A) Overview of the preparation of the Erlenmeyer flasks and the five different sources of microorganisms that were used for the propagation step; (B) Reactor R3 coming from CF before the PBS washing step; (C) Reactor R2 coming from F1 before the PBS washing step.**

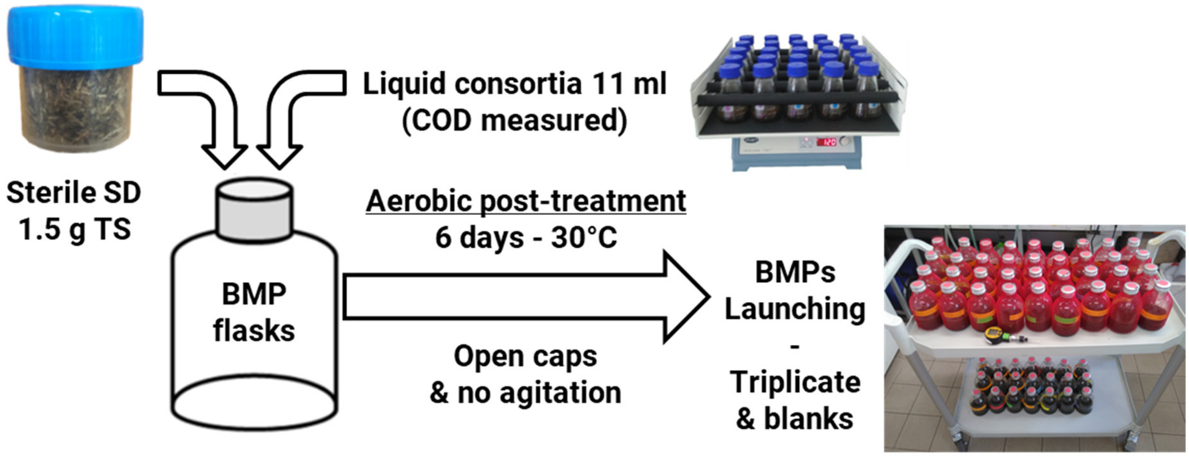

Figure S3. **Short-term aerobic post-treatment step overview**

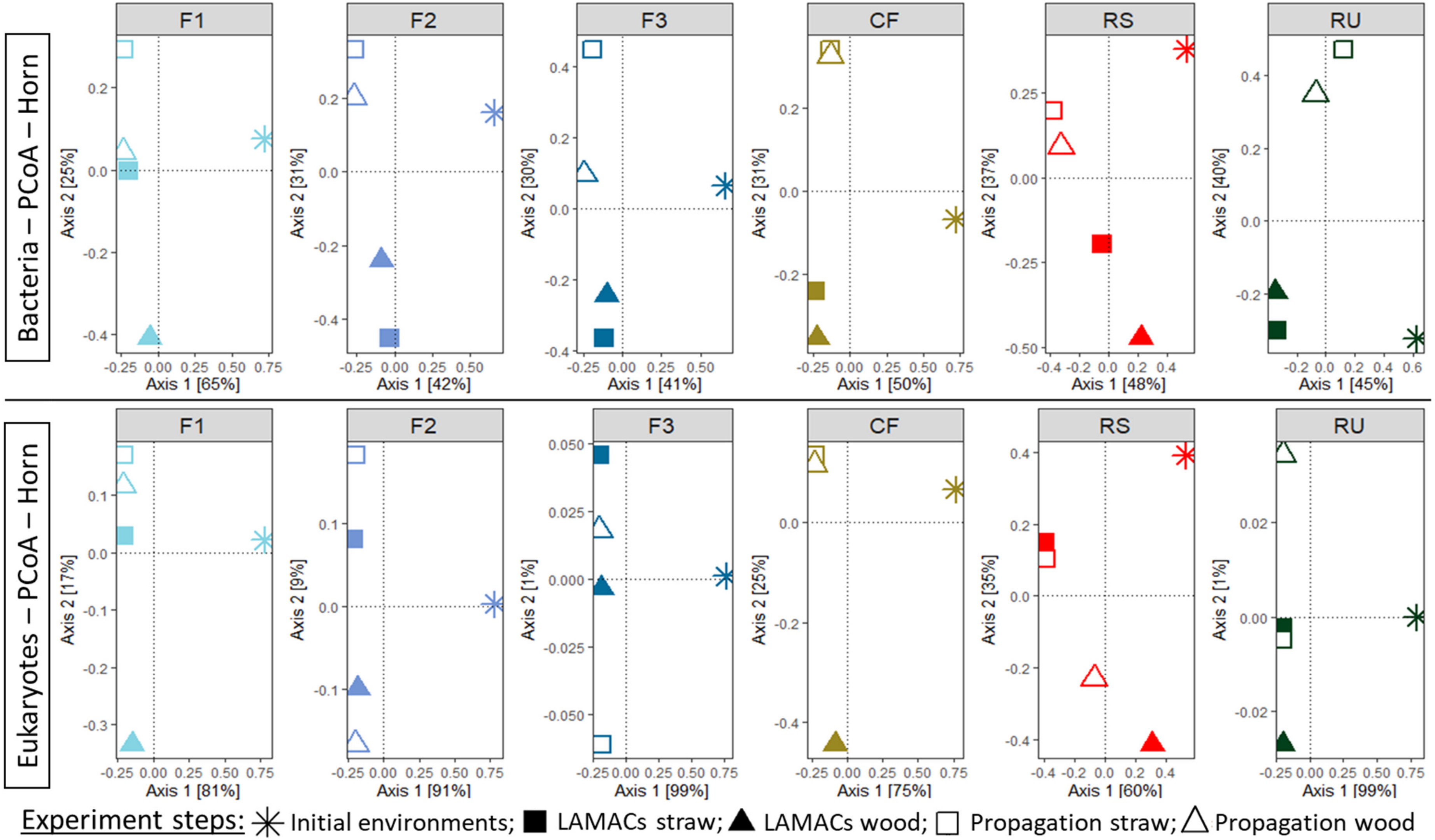

Figure S4: **Beta diversity ordination plots resulting from a Principal Coordinates Analysis (PCoA) based on Horn distance (q=1) between samples coming from a similar initial environment. Beta diversity of bacteria corresponds to the upper raw and beta diversity of eukaryotes is presented in the lower raw. A distinction is made in all these plots between the different experiment steps.**

Figure S4 displays bacteria and eukaryotes beta diversity ordination plots for the six initial environments. PCoA method was performed in the first two dimensions for all the twelve plots. Indeed, variability explained by these ordinations were considered as sufficient to withstand subsequent analysis (cumulated values are comprised between 71.2% up to 100%). Besides, PCoA has the advantage to provide non-distorted distance visualization.

In all cases, the first dimension separates the initial environments from the subsequent steps samples. For bacteria, distance between initial and subsequent samples is generally lower than for eukaryotes, as first dimension explained on average 48% of the total variance for bacteria and 84% for eukaryotes. From these observations, it can be concluded: (i) LAMACs step created an important shift in initial microbial communities due to the selective pressure applied; (ii) Higher average first dimension value for eukaryotes might be explained by a combination of a potential stronger selective pressure on eukaryotes during LAMACs step as well as a lower to absent selective pressure during propagation step, in comparison to bacteria. On that last point, no significant change in eukaryotes community composition is likely to be due to the already really low number of eukaryotes species present after the LAMACs step (on average 4 effective number of OTUs for q=1) that is unlikely to be further reduced in a liquid cultivation step.

The second dimension separates samples from LAMACs and propagation step. For bacteria, shift from LAMACs to propagation step leads to a shift in microbial communities that can be seen by an increase distance between the two groups of samples. Except for F3, distances between wood and straw samples are reduced after propagation step in comparison to LAMACs step. It means that microbial communities are getting more similar. For eukaryotes, plots with dimension 2 explaining more than 10% of total variability display identical trends (for CF plot LAMACs wood sample is located behind the propagation wood sample, explaining why it is not displayed). These distance reductions show that for most environments tested in this study, the system type (LAMACs, Erlenmeyers) drives the microbial community evolution more than the carbon sources used for selection (straw and wood).

Supplementary Methods 1

Extraction and purification

DNA from all samples analyzed in this PhD were extracted and purified using a commercial FastDNA™ SPIN Kit for Soil (MP Biomedicals, Santa Ana, United States). Manufacturer instructions were followed to recover purified DNA from all samples. The quality and quantity of DNA were verified by spectrophotometry using 2 µL of DNA extracts with a NanoQuant Plate on a Spark® microplate reader (Tecan, Männedorf, Switzerland).

Real-time quantitative polymerase chain reaction (qPCR)

The qPCR for bacteria and eukaryotes were performed in 96-well Eppendorf PCRs plates on a CFX96 thermocycler from BioRad. All samples were run following two dilutions and in triplicate to assess the technical variability associated with the measurement. PCR mixture components and PCR programs vary between bacteria and Eukaryotes qPCR:

(i) For bacteria, the following components were added: 6.5 µL Mix Biorad SsoAdvanced Universal Probes Supermix (Bio-rad, Hercules, United States), 0.5 µL of each primer and probe, 2.5 µL water and 2 µL of DNA extracts for a total volume of 12.5 µL. Primers that were used were F338-354 at 100 nM as well as R805-785 at 250 nM and the probe used was a Taqman Tamra F516-536 at 50 nM [36]. For sequence details see the table below. The bacteria qPCR was performed following a program consisting in 2 minutes at 95°C for enzyme activation followed by 40 cycles of 7 s at 95°C for dissociation and 25 s at 60°C for hybridization and elongation.

(ii) For eukaryotes, the following components were added: 12.5 µL of Bio-Rad SsoAdvanced™ Universal SYBR® Green Supermix, 1 µL of each primer, 5.5 µL water and 5 µL of DNA extracts for a total volume of 25 µL. Primers that were used were F574 at 250 nM as well as R952 at 250 nM [37]. For sequence details see the table below. The eukaryotes qPCR was also performed following a program consisting in an initial incubation of 2 min at 98 °C, 40 cycles (98 °C, 15 s; 52 °C, 30 s; 72°C, 45 s) and a final melting curve.

Finally, standard curves were generated from each assay by using 10-fold dilutions in sterilized water (Gibco, Thermo Fisher Scientific) of a target plasmid (Eurofins Genomics, Ebersberg, Germany). The average number of bacterial cells was estimated by dividing the average number of 16S rRNA gene copies per cell by a factor of 4.1 [38].

Primers and probe sequences used for the qPCR analysis

Specificity	Name	Sequences 5'-3'
Bacteria	F338-354	ACTCC TACGG GAGGC AG
	R805-785	GACTA CCAGG GTATC TAATC C
	Taqman Tamra F516-536	Yakima Yellow-TGCCA GCAGC CGCGG TAATA C-Tamra
Eukaryotes	F574	GCGGT AATTC CAGCT CCAA
	R952	TTGGC AAATG CTTTC GC

PCR amplification and sequencing

For bacteria PCR amplification, the V4-V5 regions of 16S rRNA bacterial genes were amplified with primers F515-532 (5'-GTGYC AGCMG CCGCG GTA-3') and R909-928 (5'-CCCCG YCAAT TCMTT TRAGT-3') as well as their respective linkers [39]. For eukaryotes PCR amplification, the V4 region of 18S rRNA eukaryote genes were amplified with primers F574 and R952 (detailed in **table 6**) as well as their respective linkers [37]. The PCR mixtures had a total volume of 60 µL, containing: 0.6 µL of MTP™ Taq DNA polymerase (Sigma-Aldrich), 6 µL of associated Taq buffer, 4.8 µL of Deoxynucleotide (dNTP) solution mix (GE Healthcare, Boston, United States), 1.2 µL of each primer at 300 nM, 5 µL of DNA and 41.2 µL water. In a Mastercycler thermal cycler (Eppendorf) the following PCR program was followed: after 2 min at 95°C, 30 cycles of denaturation were performed (95 °C, 60 s; hybridization was at 65 °C for bacteria and at 56°C for eukaryotes, 60 s; 72°C, 60 s), concluded by a final extension at 72°C for 10 min.

The obtained PCR products were purified and sequenced using the Illumina MiSeq kit v3 chemistry (San Diego, United States), with read lengths up to 2 × 300 bp, at the GeT PlaGe Sequencing center of the GenoToul Lifescience Network (www.genotoul.fr). Sequences were recovered after demultiplexing, cleaning and affiliating sequences using Mothur version 1.39.0, as described previously [40]. It can be highlighted that: (i) sequences that appear fewer than three times in the entire data set were removed; (ii) Alignment of the 16S rRNA and 18S rRNA sequences was done using the reference database SILVA version 128 [41]; (iii) sequences in the whole dataset were clustered into operational taxonomic units (OTUs) by an affiliation threshold of 97%.

References

36. Yu, Y.; Lee, C.; Kim, J.; Hwang, S. Group-specific primer and probe sets to detect methanogenic communities using quantitative real-time polymerase chain reaction. Biotechnol. Bioeng. 2005, 89, 670–679, doi:10.1002/bit.20347.

37. Hadziavdic, K.; Lekang, K.; Lanzen, A.; Jonassen, I.; Thompson, E.M.; Troedsson, C. Characterization of the 18s rRNA gene for designing universal eukaryote specific primers. PLoS One 2014, 9, doi:10.1371/journal.pone.0087624.

38. Klappenbach, J.A.; Saxman, P.R.; Cole, J.R.; Schmidt, T.M. rrndb: the Ribosomal RNA Operon Copy Number Database. Nucleic Acids Res. 2001, 29, 181–184, doi:10.1093/nar/29.1.181.

39. Wang, Y.; Qian, P.Y. Conservative fragments in bacterial 16S rRNA genes and primer design for 16S ribosomal DNA amplicons in metagenomic studies. PLoS One 2009, 4, doi:10.1371/journal.pone.0007401.

40. Venkiteshwaran, K.; Milferstedt, K.; Hamelin, J.; Zitomer, D.H. Anaerobic digester bioaugmentation influences quasi steady state performance and microbial community. Water Res. 2016, 104, 128–136, doi:10.1016/j.watres.2016.08.012.

41. Schloss, P.D. A high-throughput DNA sequence aligner for microbial ecology studies. PLoS One 2009, 4, doi:10.1371/journal.pone.0008230.

Supplementary Methods 2

Alpha diversity measurement

Alpha diversity measurement aims to answer the question: how diverse is a microbial sample? It can be defined as the average diversity in species composition for an individual microbial sample [42]. In this study, species correspond to the determined operational taxonomic units (OTUs) following Mothur bioinformatics pipeline. Besides, the amount of DNA sequences (also called abundance) assigned to each OTU is used to give them an importance value, that allows to distinguish rare OTUs from dominant OTUs. Construction of a phylogenetic tree, that would allow finer diversity analysis (use of phylogenetic distance between OTUs), was not performed due to technical limitations (computer calculation performance) regarding the large amount of OTUs obtained from all samples (more than a thousand) [43].

The three most commonly used alpha diversity measurements are: richness, Shannon and Simpson.

(i) Richness corresponds to the total number of OTUs present in the sample (similar weight for all OTUs);
(ii) Shannon index measures entropy, that corresponds to the incertitude in the OTU identity of a randomly selected sequence in the sample;
(iii) Simpson index yields the probability that two DNA sequences selected in a random way effectively belong to two different OTUs [44].

It has been reported that a robust estimation of microbial diversity was only possible with Shannon and Simpson measurements. Indeed, richness measurement is highly uncertain due to the difficultly to determine total number of rare species in an environment via traditional sampling [45]. However, values obtained from Shannon and Simpson diversity measurements are difficult to interpret and to compare without any mathematical transformation. Thus, Hill numbers, that gather directly these measurements (as well as richness) in a single statistical framework, has been preferred for this study. Hill numbers are always expressed in "effective number of OTUs", which can be defined as the number of equally abundant OTUs that would be required to provide a similar value of diversity [46]. This unique expression way is a major advantage of Hill numbers that greatly simplify interpretation and comparison between samples.

In the study of Alberdi et al., formulas and correspondence between the different alpha diversity measurements are presented [47]. The parameter q allows to modulate sensitivity towards rare and abundant OTUs. The higher the parameter q value, the lower the sensitivity towards rare OTUs. Besides, it can be noticed that relative abundance (i.e. a percentage of the total amount of DNA sequences measured in the sample) is used for alpha diversity measurement in general. Finally, the R package hillDiv was used to plot continuous alpha diversity curves that were obtained for q parameter values varying from 0 to 2 [15].

Beta diversity measurement

Beta diversity measurement aims to answer the question: how different are microbial samples from each other? It measures the difference in microbial composition between all samples. In the framework of Hill numbers, beta diversity is obtained by dividing gamma diversity, that can be defined as the diversity of the entire meta-community, combining all the samples, with the averaged basic sums of the samples alpha diversity. Corresponding equations are presented in Table 16. It gives a number comprised between 1 (when all samples are identical) and the total number of samples (when all samples are totally different). Finally, The Hill numbers beta diversity can be further used to measure similarity index between samples.

In this study, only the Sørensen-type overlap will be used. This dissimilarity index, quantifies the average proportion of a sample OTUs that are shared across all samples [47]. It ranges between 0 (when all compared samples are identical) and 1 (when all compared samples are totally different). Similarly, to alpha diversity, q parameter can vary between 0 and 2 and the higher its value, the higher the importance given to abundant species. For q = 0, it corresponds to the well-known Bray-Curtis index, for q = 1 it corresponds to the classical Horn index and for q = 2, it corresponds to the Morisita-Horn index [48]. As previously, hillDiv R package was used to calculate beta diversity for q=1 and principal ordinations were displayed via NMDS or PCoA methods [15].

Dominant OTUs and microbial growth over time

To display evolution of the dominant OTUs over the different experiment steps, relative abundance from sequencing results for bacteria and eukaryotes were used in the Phyloseq R package [16]. Dominant OTUs were defined as those with a relative abundance above 3%, this threshold was arbitrarily selected. PCA plotting and Envfit analysis were performed on OTU relative abundance table (variables: OTUs; samples: microbial sampling) using the Vegan R package. Significant OTUs (p value<0.01 arbitrarily selected) were blasted using NCBI Blast® platform (Rockville Pike, United States).

References

42. Legendre, P.; Legendre, L. Numerical Ecology; Elsevier, Ed.; Third Engl.; 2012; ISBN 978-0-444-53868-0.

43. Lozupone, C.; Knight, R. UniFrac : a New Phylogenetic Method for Comparing Microbial Communities. Appl. Environ. Microbiol. 2005, 71, 8228–8235, doi:10.1128/AEM.71.12.8228.

44. Chao, A.; Chiu, C.; Jost, L. Unifying Species Diversity, Phylogenetic Diversity, Functional Diversity, and Related Similarity and Differentiation Measures Through Hill Numbers. Annu. Rev. Ecol. Evol. Syst. 2014, 45, 297–324, doi:10.1146/annurev-ecolsys-120213-091540.

45. Haegeman, B.; Hamelin, J.; Moriarty, J.; Neal, P.; Dushoff, J.; Weitz, J.S. Robust estimation of microbial diversity in theory and in practice. ISME J. 2013, 7, 1092–1101, doi:10.1038/ismej.2013.10.

46. Jost, L. Partitioning diversity into independant alpha and beta components. Ecology 2007, 88, 2427–2439, doi:10.1890/06-1736.1.

47. Alberdi, A.; Gilbert, M.T.P. A guide to the application of Hill numbers to DNA-based diversity analyses. Mol. Ecol. Resour. 2019, 19, 804–817, doi:10.1111/1755-0998.13014.

48. Chao, A.; Chiu, C.H. Bridging the variance and diversity decomposition approaches to beta diversity via similarity and differentiation measures. Methods Ecol. Evol. 2016, 7, 919–928, doi:10.1111/2041-210X.12551.
